# Urethral Lift as a Safe and Effective Procedure for Prostatic Hyplasia Population: A Systematic Review and Meta-Analysis

**DOI:** 10.3389/fsurg.2020.598728

**Published:** 2020-12-08

**Authors:** Jibo Jing, Yuqing Wu, Mulong Du, Nieke Zhang, Meiling Wang, Bin Xu, Ming Chen

**Affiliations:** ^1^Department of Urology, Affiliated Zhongda Hospital of Southeast University, Nanjing, China; ^2^Surgical Research Center, Institute of Urology, Medical School of Southeast University, Nanjing, China; ^3^Lishui People's Hospital, Nanjing, China; ^4^Jiangsu Key Laboratory of Cancer Biomarkers, Prevention and Treatment, Department of Environmental Genomics, Collaborative Innovation Center for Cancer Personalized Medicine, Nanjing Medical University, Nanjing, China; ^5^Department of Biostatistics, Center for Global Health, School of Public Health, Nanjing Medical University, Nanjing, China

**Keywords:** prostatic urethral lift (PUL), urolift, BPH (benign prostatic hyperplasia), LUTS (lower urinary tract symptoms), PUL

## Abstract

**Background:** Prostatic urethral lift (PUL) is a relatively new minimally invasive treatment procedure for benign prostatic hyperplasia (BPH). In order to analyze the sustainability of this new protocol, a systematic review and meta-analysis is performed based on the published articles.

**Methods:** We performed a critical review according to the preferred reporting items for systematic review and meta-analysis (PRISMA) and MOOSE guidelines. A total of 818 published articles matched our search terms, and 11 studies met the inclusion criteria. Data of each follow-up time point (1, 3, 6, 12, and 24 months) were analyzed in terms of baseline characteristics and functional and sexual health outcomes. The merged means of each time point were calculated using R package meta and shown in the tendency plot.

**Results:** A total of 1,443 patients who underwent PUL are available for the research. At 24 months, the changes of the three indicators are statistically significant (IPSS 9.40 points, *p* < 0.001; Qmax 3.39 ml/s, *p* < 0.001; QoL 1.99 points, *p* < 0.001) but were not as effective as TURP (from literature). The trend plot shows that, as time goes on, the effect of PUL tends to increase first and then weaken. Three items fitted a meaningful curve: IPSS (slope = −1.378 *t* = −12.395, *p* < 0.001), Qmax (slope = −1.382 *t* = −6.429, *p* < 0.001), and QoL (slope = −0.218, *t* = −10.058, *p* < 0.001). Fitted curves of SHIM and PVR are not statistically significant. The regression reveals that IPSS, Qmax, and QoL could be predicted after accepting PUL.

**Conclusion:** PUL appears to be a safe and effective procedure in selected patients with BPH and can improve the symptoms of urinary tract obstruction. However, it is not as effective as TURP and shows no influence to the objective indicators like PVR.

## Introduction

Benign prostatic hyperplasia (BPH) is one of the most common conditions of the aging male, and it usually presents with lower urinary tract symptoms (LUTS). With an aging population, the prevalence will rise further, thus resulting in higher medical costs and heavier burden on healthcare resources.

Treatment of BPH includes both medical and surgical management. About 30% patients chose surgical management after medical management because of the unsatisfactory response, side effects, cost, and difficulty with compliance, sexual dysfunction, or a combination of reasons regarding drug therapy (1, 2). Currently, transurethral urethral resection of prostate (TURP) remains the gold standard procedure for BPH. However, about 53–75% of patients undergoing TURP are at the risk of retrograde ejaculation, which impacts the patients' quality of life (3). Complication including erectile dysfunction (2–10%), incontinence (2–10%), and retrograde ejaculation (65–75%) limits its clinical application (4, 5).

Prostatic urethral lift (PUL) is a minimally invasive surgery that uses implants to push the lateral lobes of the prostate to minimize the adverse effects of traditional TURP (6). Early reported outcomes of procedures like PUL have no sexual side effects, and it can be performed under local anesthesia. It could be a superior choice for patients with poor cardiopulmonary function.

We reviewed the research that evaluated the effectiveness of PUL and collected and synthesized the data to perform a systematic review, starting with three items for tract symptoms and bladder function, International Prostate Symptom Score (IPSS), peak flow rate (Qmax), and post-void residual volume (PVR). The quality of life index (QoL) and Sexual Health Inventory for Men (SHIM) were used to assess the effectiveness of PUL in easing BPH symptoms and its influence on sexual function and life quality.

## Materials and Methods

The research was performed in accordance with MOOSE and PRISMA 2009 guidelines (4, 5).

### Search Strategy

A systematic literature search was conducted in the PubMed, Web of Science, and Cochrane library for English language studies before December 1, 2019. The following keywords were used:

1. “PUL” OR “urethral lift”.
2. “Benign prostatic hypertrophy” OR “BPH” OR “Benign prostatic hyperplasia” OR “lower urinary tract symptoms” OR “LUTS”.
3. “prognosis” OR “Urination symptoms” OR “prostate symptoms” OR “follow up” OR “PVR” OR “SHIM” OR “IPSS” OR “Qmax” OR “Qol”.

Full text search was applied.

### Inclusion and Exclusion Criteria

Inclusion criteria are as follows:

1. Clinical study (array research, RCT) that focused on the efficacy of PUL in BPH population.
2. The prostate symptom indicators are provided in the paper (IPSS, QoL, and at least one of the following data: Qmax, PVR, or SHIM).
3. Patients were diagnosed with BPH due to lower urinary tract symptoms. The diagnosis criteria of BPH should be uniformed.
4. The subjective parts in evaluating the symptom indicators like IPSS should be completed by at least two people.

Exclusion criteria are as follows:

1. Review, writing, and commentary studies with unavailable data or insufficient level of evidence, etc.
2. Absence of specific data, and unavailability of data even after trying to contact the original author.

### Methods of Review

Two authors independently screened the studies for eligibility, and disagreements were judicially resolved by the third reviewer. The data extraction and critical appraisal were also carried out independently by two reviewers.

We used the Newcastle–Ottawa Quality Assessment Scale for cohort studies (NOS) (7) to evaluate the quality of the non-randomized studies. This scale contains eight elements to assess patient population and selection, study comparability, follow-up, and outcome of interest. High-quality elements are awarded by adding a star, and then the stars are added up to compare the study quality. Each study was graded as either low quality (0–5) or high quality (6–9), and low-quality studies were excluded. The results are presented in Table 2.

### Research Design

Firstly, the change of each index (IPSS, QoL, Qmax, PVR, and SHIM) after the patient underwent PUL was compared with the baseline value. Then, compare each index between patients who have undergone PUL and TURP. The trend graphs of the changes in each index after the PUL procedure were made in an attempt to clarify the effectiveness of the PUL procedure. For indicators for which data were not available in the study due to the long follow-up period, comparisons were performed with data from guidelines or reviews.

### Statistical Analysis

All statistical calculations were completed by R (3.6.1). All collected data are processed as continuous variable (mean, SD). We collected the symptom index data at every time point (1, 3, 6, 12, and 24 months) after the patients accepted the PUL (i.e., IPSS, Qmax, PVR, QoL, and SHIM). Using package meta, function metamean to calculate the merged mean (inverse variance weighted model), *I*^2^, and 95% confidence intervals (CI) were determined. When a study reported a range instead of standard deviation (SD), we converted the extremum to SD using the method from Rassweiler et al. (3) and then drew the tendency plot. Random effects models were applied owing to the high heterogeneity (*I*^2^ > 50%) of the studies. Otherwise, fixed-effects model was used (4, 6). Finally, box plot and time-regression plot were drawn according to forest plot data and merged means data to show the whole tendency.

Funnel plots and Egger's linear regression test were conducted to assess the publication bias (8) with STATA (version15.0, Stata Corp LP, College Station, TX). A value of *p* < 0.05 was considered significant.

## Result

### Search Results

**Figure 1:**
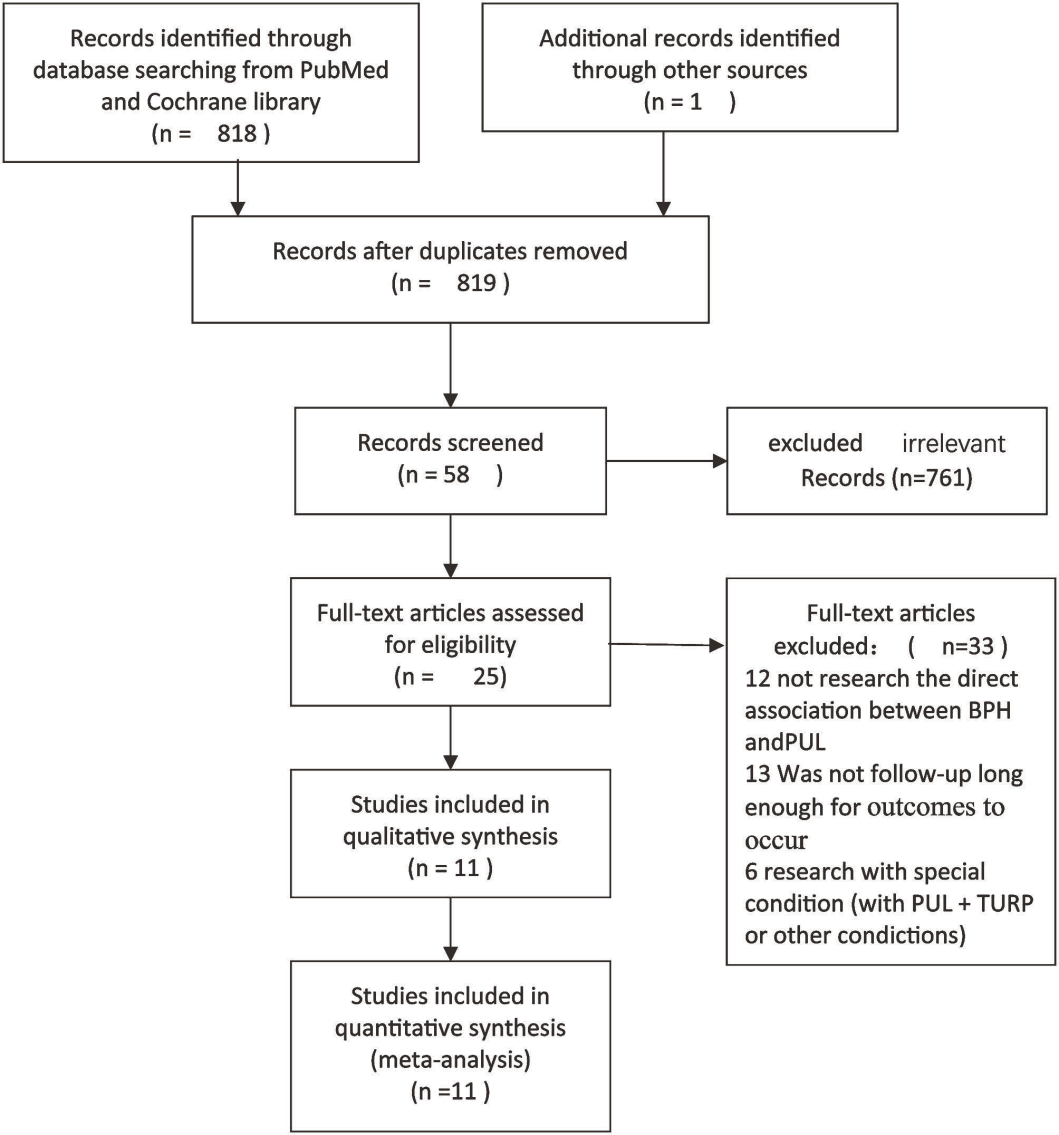
Flowchart.

A total of 819 non-repetitive studies were obtained from PubMed, Web of Science, and Cochrane Library, and 761 unrelated studies were excluded. A total of 33 unrelated studies were removed by reading the title and abstract. Twenty-five studies entered the full-text stage to assess eligibility. Finally, 11 articles were selected to extract data, and all of them are array research. Publication time ranges from 2012 to 2018. A total of 1,443 patients who underwent PUL are available for the researches. Screen flow chart is shown in Figure 1. The result of NOS scale is shown in Table 1 (9–19).

**Table 1:** The result of NOS rank.

**Reference**	**Selection**				**Comparability**	**Outcome**			**Total**
	**1**	**2**	**3**	**4**		**1**	**2**	**3**	
Woo et al. (9)	*	*	*	*	*	*	*		7
Sønksen et al. (10)	*	*	*	*		*	*		6
Sievert et al. (11)	*	*	*	*	*	*	*	*	8
Rukstalis et al. (12)	*	*	*	*		*	*	*	7
Rukstalis et al. (13)	*	*	*	*	*	*	*		7
Roehrborn et al. (14)	*	*	*	*	*	*	*	*	8
McVary et al. (15)	*	*	*	*		*	*		6
Eure et al. (16)	*	*	*	*	*	*	*	*	8
Chin et al. (17)	*	*	*	*		*	*	*	7
Cantwell et al. (18)	*	*	*	*	*	*	*		7
Bozkurt et al. (19)	*	*	*	*		*	*		6

### Forest Plot of Symptoms (Baseline vs. Follow-Up Point)

**Figure 2:**
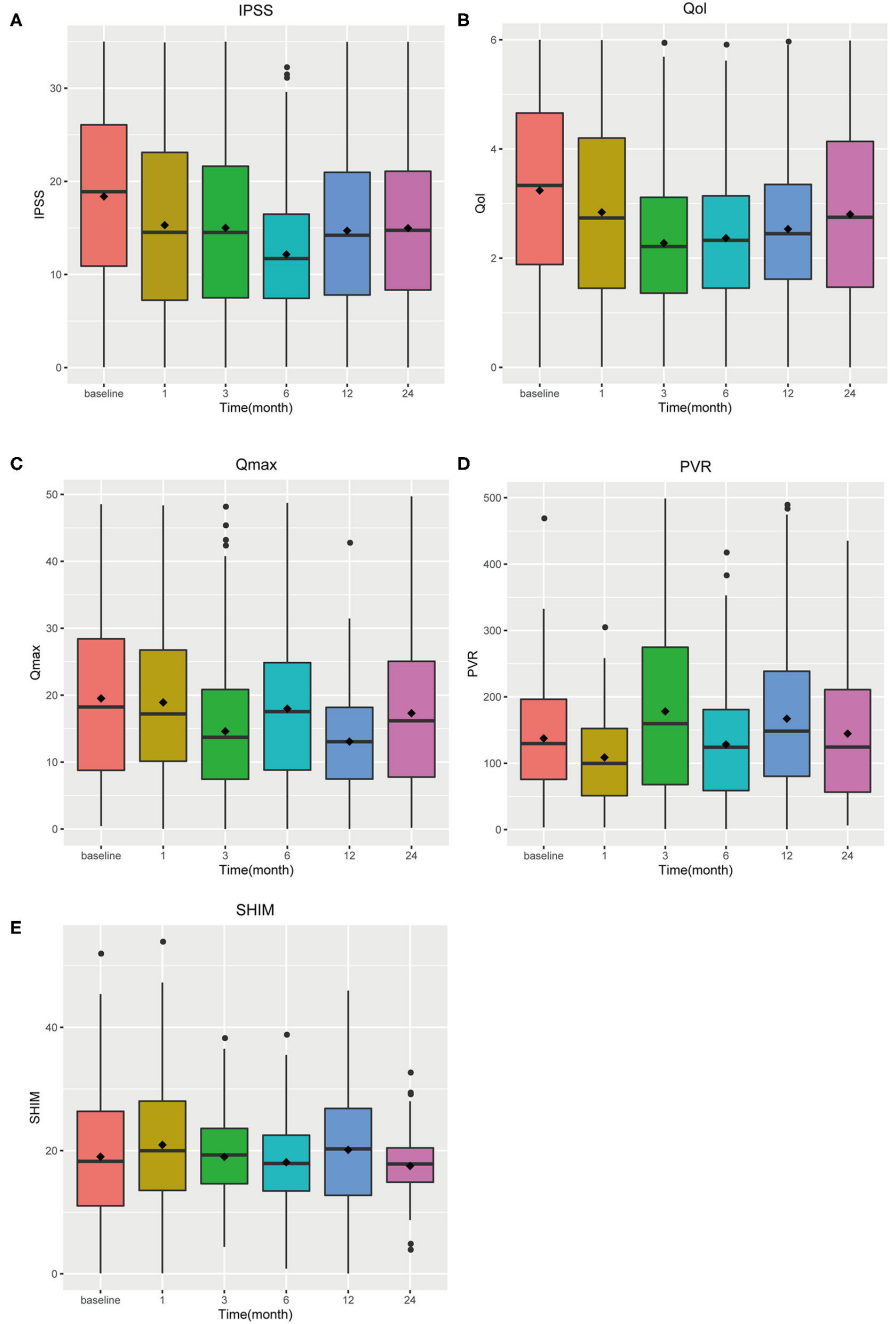
Box plot of tendency **(A)** IPSS **(B)** QoL **(C)** Qmax **(D)** PVR **(E)** SHIM.

Data of five observed items (IPSS, QoL, Qmax, PVR, and SHIM) were collected (Supplementary Tables 2–6). Then, we drew a forest plot of each time point vs. the corresponding baseline. Data of 25 forest plots are shown in Supplementary Tables 7–11. IPSS, Qmax, and QoL show significant improvement in all follow-up time points (*p* < 0.001). PVR and SHIM have a statistical difference only in the 3rd month after PUL (*p* = 0.011 for PVR and *p* = 0.022 for SHIM). According to the box plot (Figures 2A–E), IPSS, Qmax, and QoL show an aggravating tendency, and no clear trend was shown in the other two items.

### LUTS and Bladder Function Improved

**Figure 3:**
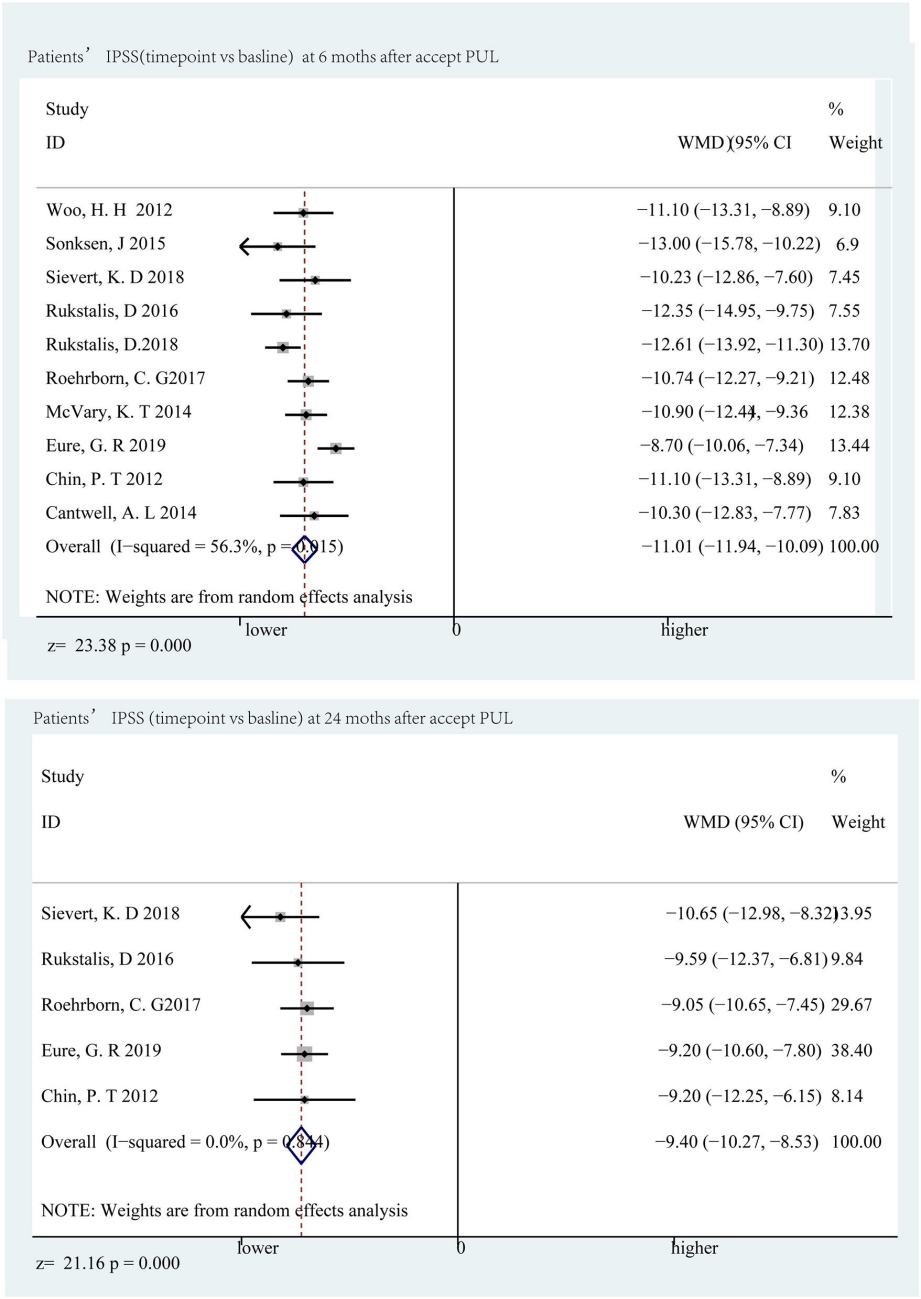
Forrest plot of IPSS (follow-up time point vs. baseline) 6th and 24th months.

**Figure 4:**
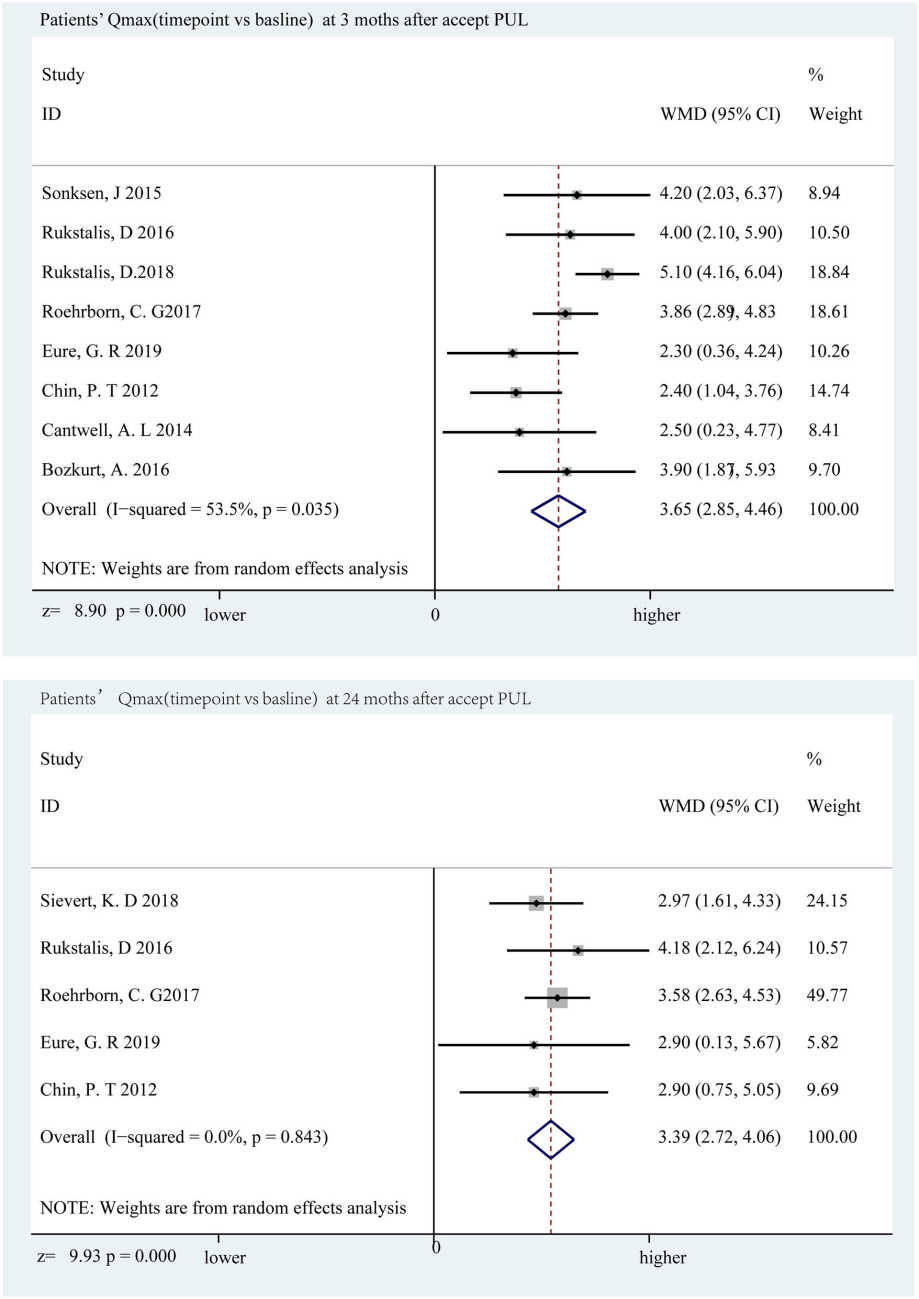
Forrest plot of Qmax (follow-up time point vs. baseline) 3rd and 24th months.

**Figure 5:**
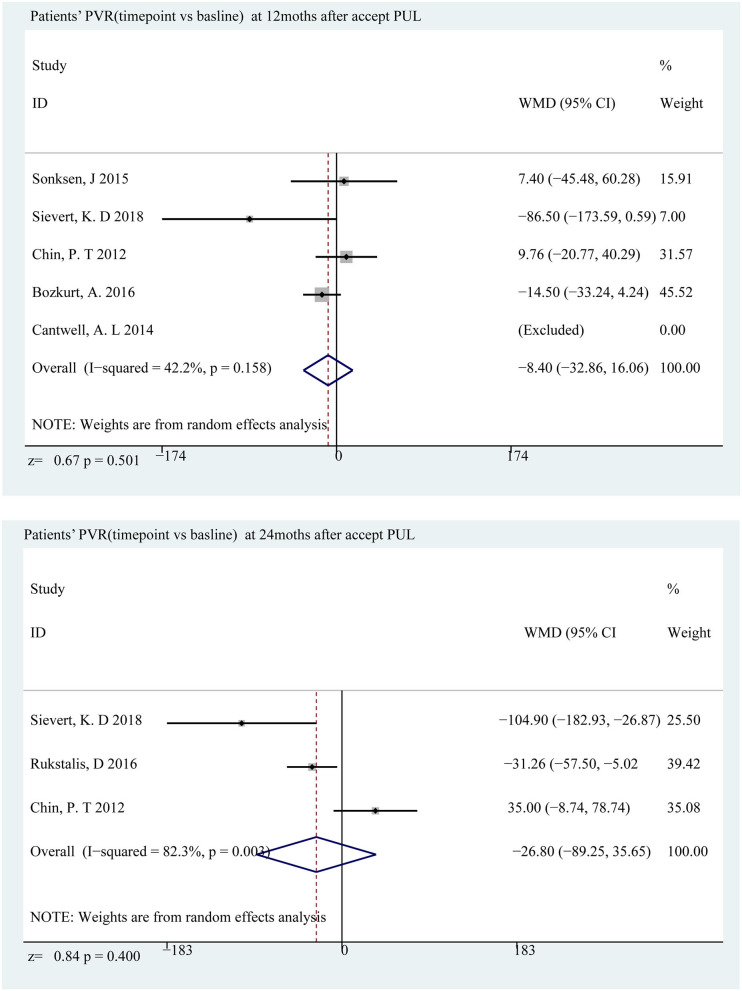
Forrest plot of PVR (follow-up time point vs. baseline) 12th and 24th months.

The decline in IPSS was most distinct at the sixth month, which was a decrease of 11.01 points (*p* < 0.001) compared to the baseline, and a significant decrease of 9.40 points (*p* < 0.001) (Figure 3) appeared below the baseline at the 24th month. Qmax and PVR can reflect bladder function, and Qmax is expected to have the largest difference in the third month, increasing by 3.65 ml/s (*p* < 0.001) and 3.39 ml/s (*p* < 0.001) at the 24th month (Figure 4); however, there was almost no change in PVR during follow-up (Figure 5). Merged means calculated by R (3.6.0) shows that IPSS and Qmax have a clear decline trend in the first 6 months after patients accepted PUL, but slowly recover in the remaining follow-up time (Figures 2A,C and Supplementary Tables 11, 13). PVR has no clear tendency (Figure 2D and Supplementary Table 14). According to a prospective research, 6 and 18 months after accept monopolar TURP, patients' IPSS decreased to 7.6 and 8.3 points, which is relatively lower than PUL (10.94 points at the 6th month and 12.08 at the 24th month). Similarly, after undergoing TURP surgery, patients' Qmax were 20.6 and 20.2 ml/s at the 6 and 18th months, respectively; data of PUL patients were 12.310 and 12.615 ml/s in this study. PVR changed to 24 and 33 ml, and 52.910, and 57.050 ml in this study (20).

### Sexual Function and Life Quality

**Figure 6:**
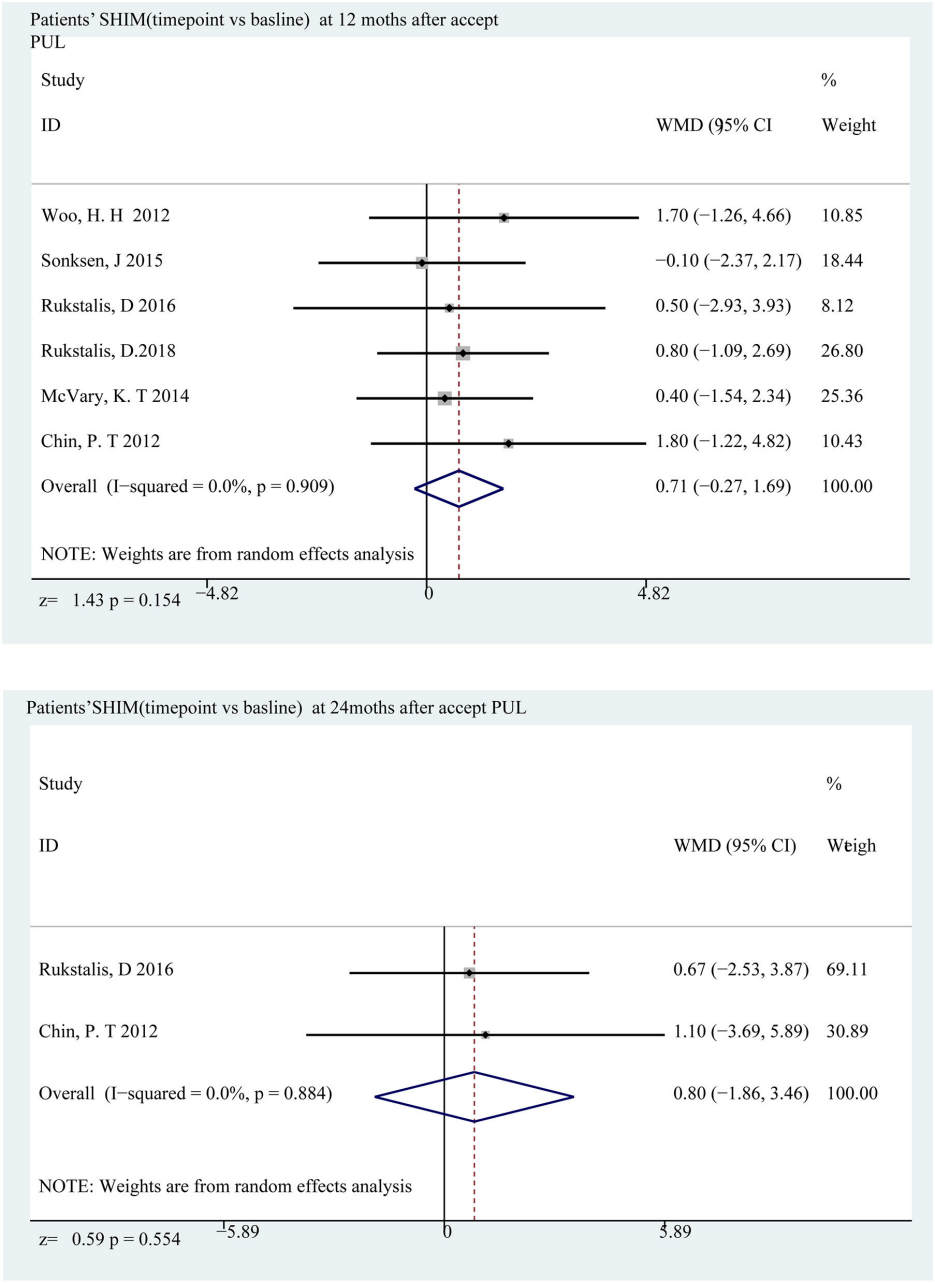
Forrest plot of SHIM (follow-up time point vs. baseline) 12th and 24th months.

**Figure 7:**
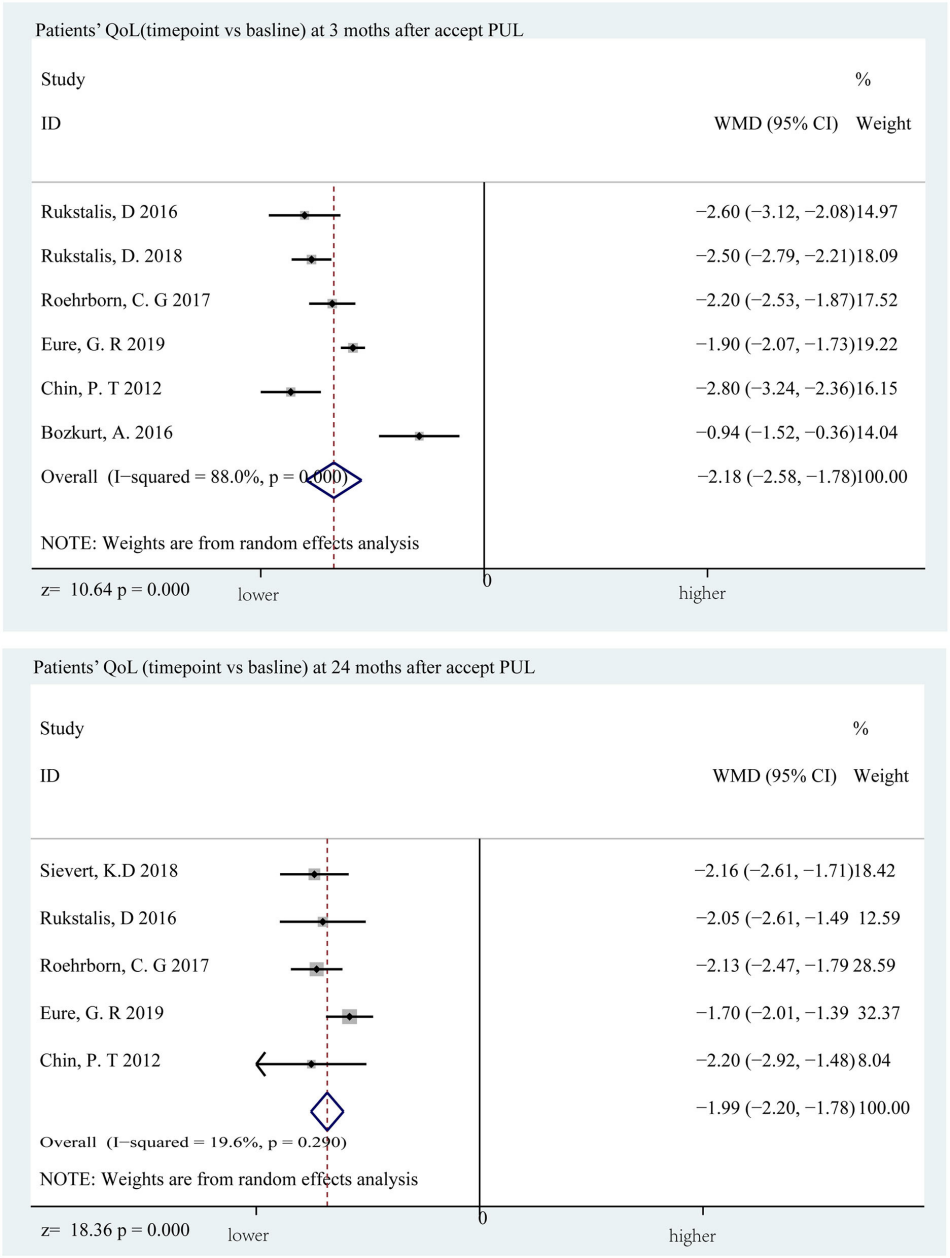
Forrest plot of QoL (follow-up time point vs. baseline) 3rd and 24th months.

The sexual function evaluation index was meaningless at almost the entire follow-up period. At the 12 and 24th months, the changes from baseline were 0.710 points (*p* = 0.154) and 0.803 points (*p* = 0.554) (Figure 6), indicating that PUL has no effect on patients' sexual function. When it comes to quality of life, there are some differences. The difference is most obvious at the 3rd month, with an average reduction of 2.176 points (*p* < 0.001) and lower than the baseline value at the end of the follow-up (1.99 points, *p* < 0.001) (Figure 7). Merged mean box plot of SHIM reveals no tendency (Figure 2E and Supplementary Table 15), QoL has a similar decline trend to IPSS (Figure 2B and Supplementary Table 12) compared to TURP, and QoL is 1.3 and 1.5 at the 6 and 18th month, which are better than PUL's 2.208 (6th month) and 2.382 (24th month) (20).

### Regression of Merged Mean Data

**Figure 8:**
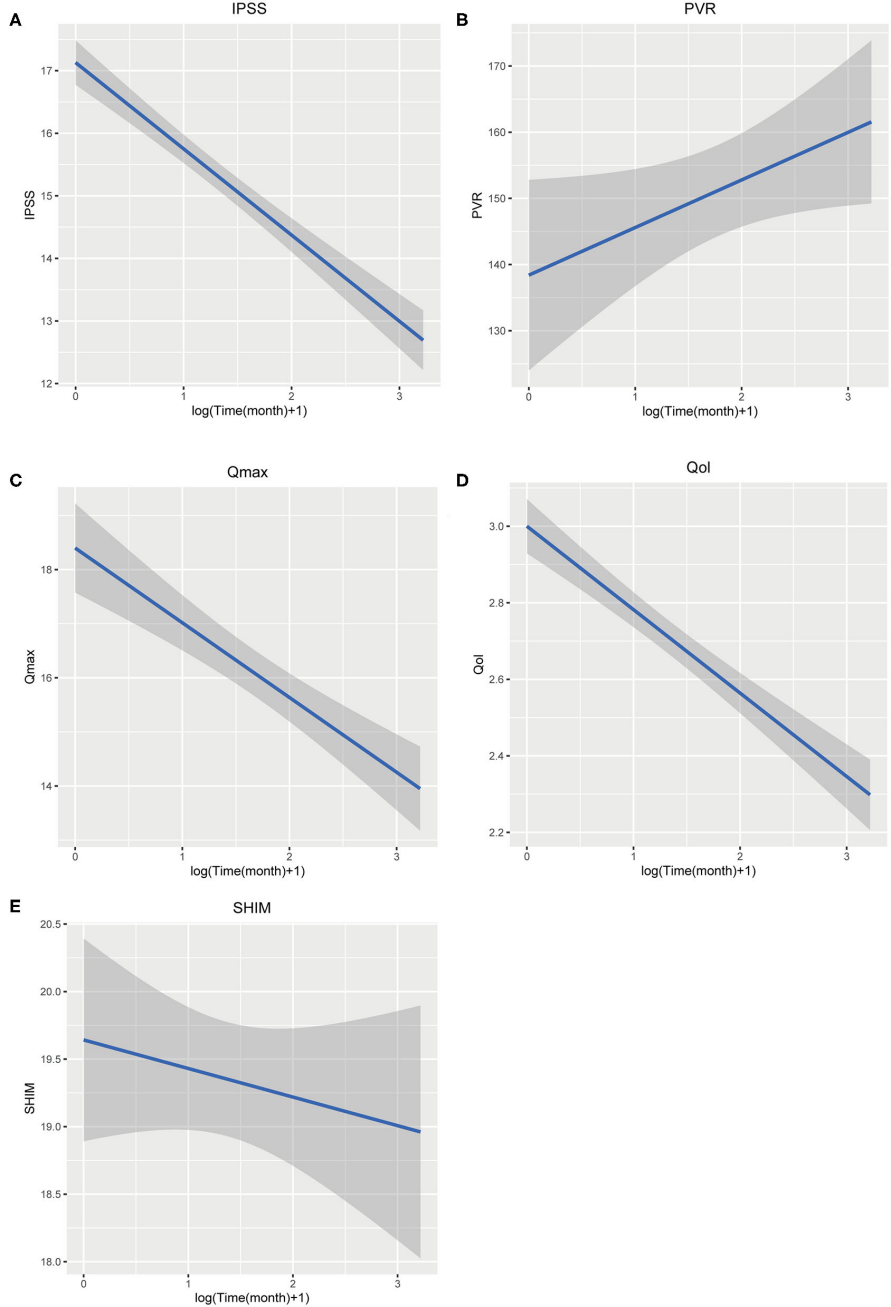
**(A–E)** Liner regression of merged means.

In order to quantify the change tendency, we perform linear regression based on the merged means, with Log [Time(month+1)] as the *X* axis and target observation items as the *Y* axis (Figures 8A–E). The findings are summarized in Table 2. IPSS and Qmax have a clear tendency, coefficient IPSS of is −1.378 (*t* = −12.395, *p* < 0.001), and Qmax's slope is −1.382 (*t* = −6.429, *p* < 0.001). QoL also could fit meaningful curves (slope = −0.218, *t* = −10.058, *p* < 0.001). Fitted curves of SHIM and PVR are not statistically significant (Table 2). The regression reveals that IPSS, Qmax, and QoL could be predicted after accepting PUL.

**Table 2:** Data of merged mean regression.

**Variable**	**Estimate**	**Std.error**	***t-*value**	**Pr**
IPSS	−1.378	0.112	−12.359	<0.001
QoL	−0.218	0.022	−10.058	<0.001
Qmax	−1.382	0.215	−6.429	<0.001
SHIM	−0.212	0.231	−0.918	0.359
PVR	7.193	3.62	1.987	0.047

## Discussion

Most previous studies have suggested that PUL can significantly improve the symptoms and quality of life of LUTS (9, 10, 21, 22); only a small number of studies believe that this improvement is not significant compared to TURP (23–26). The above results show that PUL has improved the symptoms of BPH to a certain degree, but still has little difference compared to the former researches.

First, PUL can significantly ease the symptoms of LUTS caused by BPH and ameliorate the IPSS to moderate symptoms (8–19). The average decrease of 11.1 points is similar to the previous study. However, this improvement trend slightly weakens after 6 months; a 9.40-point decrease occurs compared to the baseline (2–4 points) at the 24th month. This suggests that patients undergoing PUL should start regular review after 6 months. The quality of life has a slight decline in the whole follow-up period, but it is not so significant according to the clinical practice and is still between satisfaction and dissatisfaction (3 points). These two results suggest that PUL can improve the symptoms of lower urinary tract obstruction to some extent, but it cannot significantly improve the quality of life of patients (although there are statistical differences, according to the quality of life index, the difference is not clinically significant).

The data fitting results show that the changes in IPSS and QoL follow a certain linear relationship, which can be used to predict the improvement of symptoms after surgery. Curves of Qmax are also significant. Calculating according to the fitted regression curve, we found that the effect of PUL slowly decreased after surgery and eventually stabilized.

Objective indicators, like PVR, which can best reflect the degree of obstruction, were normal at the beginning (>15 ml). During the follow-up process, these indicators showed no signs of improvement, and even two decreasing values appeared. The PVR that reflects the ability of the bladder detrusor suggests that the vast majority of patients have been in an irreversible decompensation (PVR > 100 ml) before receiving PUL. Therefore, the patients' PVR did not present clinically meaningful changes after surgery.

In terms of side effects, the same as previous research conclusions, the SHIM scale showed that the patient's sexual function did not change significantly and maintained a high degree of consistency within 24 months of follow-up.

Our study quantitatively analyzes the effectiveness of PUL for LUTS caused by BPH in the application of evidence-based medicine. The application of statistical tools more intuitively draws conclusions that are relatively different from previous studies. It can be seen from these data that although PUL has improved the LUTS of patients to some extent, it still has a certain gap with TURP.

Here, we focus on a newly published article with the same subject as our research (27–30), and some of the results differ from our research. This study analyzed the effectiveness of PUL by comparing the gap between the follow-up value at 24 months and the baseline value. The authors have shown that PUL has a significant effect on improving LUTS symptoms and signs without affecting sexual function and quality of life. However, the data applied by the author to prove his conclusions are relatively small and insufficient, which leads to a lack of persuasiveness. The author only compares the results of the 24th month, so they did not notice that there is a linear relationship between time after accepting PUL and LUTS; that is, PUL has a relatively better effect in the initial period (3–6 months), and this effect would reduce over time. In addition, we noticed that this study may have made a small mistake. In the study of PVR, incorrect baseline values may be included, which biased the results toward the positive side.

## Conclusion

PUL appears to be a safe and effective procedure in selected patients with BPH and can improve the symptoms of urinary tract obstruction. However, it is not as effective as TURP and cannot improve objective indicators like PVR.

## Data Availability Statement

The original contributions presented in the study are included in the article/Supplementary Materials, further inquiries can be directed to the corresponding author/s.

## Author Contributions

JJ and BX conceived of the presented idea. MC developed the theory and performed the computations. JJ and MD verified the analytical methods. YW encouraged JJ to investigate IPSS and supervised the findings of this work. All authors discussed the results and contributed to the final manuscript.

## Conflict of Interest

The authors declare that the research was conducted in the absence of any commercial or financial relationships that could be construed as a potential conflict of interest.
