# Supplementary material for: Urethral Lift as a Safe and Effective Procedure for Prostatic Hyplasia Population: A Systematic Review and Meta-Analysis
Source: Front Surg. 2020 Dec 8;7:598728. doi: 10.3389/fsurg.2020.598728 (PMC7793831; doi:10.3389/fsurg.2020.598728)
Supplement: Supplementary file 1 [file Data_Sheet_1.zip › Data Sheet 1.DOCX]

**Supplement Table. 1** Newcastle–Ottawa quality assessment scale

| **Selection** |
| --- |
| (1) Representativeness of the exposed cohort |
| (a) Truly representative of the average ‘‘BPH patient’’in the community (1 star) |
| (b) Somewhat representative of the average ‘BPH patient’’in the community (1 star) |
| (c) Selected group of users (e.g., nurses, volunteers) |
| (d) No description of the derivation of the cohort |
|  |
| (2) Selection of the non-exposed cohort |
| (a) Drawn from the same community as the exposed cohort(1 star) |
| (b) Drawn from a different source |
| (c) No description of the derivation of the non-exposed cohort |
|  |
| (3) Ascertainment of exposure |
| (a) Secure record (e.g., surgical records) (1 star) |
| (b) Structured interview (1 star) |
| (c) Written self-report |
| (d) No description |
|  |
| (4) Demonstration that outcome of interest was not present at start of study |
| (a) Yes (1 star) |
| (b) No |
|  |
| **Comparability** |
| (1) Comparability of cohorts on the basis of the design or analysis |
| (a) Study controls for ‘‘age, sex, BMI’’ (1 star) |
| (b) Study controls for any additional factor (1 star) (ASA, tumorsize, stage etc.) |
|  |
| **Outcome** |
| (1) Assessment of outcome |
| (a) Independent blind assessment (1 star) |
| (b) Record linkage (1 star) |
| (c) Self-report |
| (d) No description  (2) Was follow-up long enough for outcomes to occur?  (a) Yes (‘‘2 years’’) (1 star)  (b) No  (3) Adequacy of follow-up of cohorts  (a) Complete follow-up—all subjects accounted for (1 star)  (b) Subjects lost to follow-up unlikely to introduce bias—small number lost ‘‘5 %’’ or description provided of those lost (1 star)  (c) Follow-up rate ‘‘[95 %’’ and no description of those lost  (d) No statement |

a A study can be awarded a maximum of one star for each numbered item within the Selection and Outcome categories. A maximum of two stars can be given for comparability. Underlined and quoted phrases are provided in the scale to allow for adjustment to particular studies. Italicized phrases indicate our interpretation of the question relevant to this study
