# Supplementary material for: Urethral Lift as a Safe and Effective Procedure for Prostatic Hyplasia Population: A Systematic Review and Meta-Analysis
Source: Front Surg. 2020 Dec 8;7:598728. doi: 10.3389/fsurg.2020.598728 (PMC7793831; doi:10.3389/fsurg.2020.598728)
Supplement: Supplementary file 1 [file Data_Sheet_1.zip › Data Sheet 2.DOCX]

Supplement table 2 IPSS(mean,SD)

| Reference | 1m | | | 3m | | | 6m | | | 12m | | | 24m | | |
| --- | --- | --- | --- | --- | --- | --- | --- | --- | --- | --- | --- | --- | --- | --- | --- |
|  | N | Follow-up | Baseline | N | Follow-up | Baseline | N | Follow-up | Baseline | N | Follow-up | Baseline | N | Follow-up | Baseline |
| Woo, H. H 2012 | 62 | 8.6,5.4* | 22.7,5.3 | 62 | 9.1,5.1 | 22.7,5.3 | 62 | 11.6,7.1 | 22.7,5.3 | 55 | 12.1,7.1 | 22.5,5.4 |  |  |  |
| Sonksen, J 2015 | 44 | 10.5,7.6 | 22.1,5.7 | 42 | 10.5,7.4 | 22.3,5.8 | 44 | 9.2,7.5 | 22.2,5.7 | 32 | 10.7,8.1 | 22.0,5.6 |  |  |  |
| Sievert, K. D 2018 | 48 | 11.92,7.13 | 20.82,6.52 |  |  |  | 41 | 10.59,5.61 | 20.82,6.52 | 42 | 10.29,4.70 | 20.82,6.52 | 41 | 10.17,3.93 | 20.82,6.52 |
| Rukstalis, D 2016 | 51 | 12.32,8.01 | 25.44,5.53 | 51 | 13.06,7.71 | 25.41,5.48 | 51 | 13.06,7.71 | 25.41,5.53 | 49 | 15.22,8.14 | 25.49.5.58 | 42 | 15.17,7.2 | 24.76,5.69 |
| Rukstalis, D.2018 | 180 | 11.7,6.7 | 22.7,5.4 | 181 | 10.4,7.2 | 22.8,5.4 | 178 | 10.09,7.1 | 22.7,5.4 | 167 | 11.3,7.2 | 22.7,5.5 |  |  |  |
| Roehrborn,C.G2017 | 140 | 12.43,6.92 | 22.32,5.45 | 140 | 11.26,7.56 | 22.32,5.42 | 140 | 11.58,7.45 | 22.32,5.42 | 140 | 12.36,7.51 | 22.32,5.42 | 140 | 13.27,8.0 | 22.32,5.42 |
| McVary, K. T 2014 | 137 | 12.3,6.9 | 22.1,5.4 | 137 | 11.0,7.6 | 22.1,5.4 | 133 | 11.0,7.3 | 21.9,5.4 | 123 | 11.1,7.0 | 21.8,5.4 |  |  |  |
| Eure, G. R 2019 | 666 | 11.2,5.4 | 21.3,5.4 | 427 | 11.2,5.2 | 21.1,5.2 | 193 | 10.4,6.8 | 19.1,6.8 | 204 | 11.8,5.6 | 21.0,5.6 | 131 | 12.0,5.8 | 21.2,5.8 |
| Chin, P. T 2012 | 62 | 8.6,5.4* | 22.7,5.3 | 62 | 9.1,5.1 | 22.7,5.3 | 62 | 11.6,7.1 | 22.7,5.3 | 55 | 12.1,7.1 | 22.5,5.4 | 33 | 12.6,7.2 | 21.8,5.3 |
| Cantwell,A. L 2014 | 53 | 12.5,7.0 | 23.3,5.5 | 52 | 12.3,7.9 | 23.4,5.5 | 53 | 13.0,7.6 | 23.3,5.5 | 48 | 14.6,7.7 | 23.3,5.6 |  |  |  |
| Bozkurt, A. 2016 |  |  |  | 17 | 13.3,2.5 | 22.8,4.37 |  |  |  | 17 | 13.2,2.6 | 22.8,4.37 |  |  |  |

Supplement table 3 QoL

| Reference | 1m | | | 3m | | | 6m | | | 12m | | | 24m | | |
| --- | --- | --- | --- | --- | --- | --- | --- | --- | --- | --- | --- | --- | --- | --- | --- |
|  | N | Follow-up | Baseline | N | Follow-up | Baseline | N | Follow-up | Baseline | N | Follow-up | Baseline | N | Follow-up | Baseline |
| Sievert, K. D 2018 | 45 | 2.22,1.38 | 4.14,1.22 |  |  |  | 42 | 2.05,1.10 | 4.14,1.22 | 38 | 2.21,1.38 | 4.14,1.22 | 44 | 1.98,0.90 | 4.14,1.22 |
| Rukstalis, D 2016 | 51 | 2.35,1.49 | 4.80,1.08 | 50 | 2.18,1.53 | 4.78,1.07 | 50 | 2.18,1.53 | 4.78,1.07 | 51 | 2.45,1.43 | 4.80,1.08 | 49 | 2.73,1.68 | 4.78,1.09 |
| Rukstalis, D. 2018 | 180 | 2.4,1.6 | 4.7,1.0 | 181 | 2.2,1.7 | 4.7,1.0 | 178 | 2.1,1.6 | 4.7,1.0 | 167 | 2.2,1.5 | 4.7,1.0 |  |  |  |
| Roehrborn,C.G2017 | 140 | 2.62,1.68 | 4.62,1.05 | 140 | 2.42,1.72 | 4.62,1.05 | 140 | 2.22,1.70 | 4.62,1.05 | 140 | 2.43,1.70 | 4.62,1.05 | 140 | 2.49,1.74 | 4.62,1.05 |
| Eure, G. R 2019 | 532 | 2.1,1.8 | 4.2,1.8 | 363 | 2.1,1.2 | 4.0,1.2 | 164 | 2.3,1.2 | 4.2,1.2 | 162 | 2.4,1.2 | 4.2,1.2 | 98 | 2.5,1.1 | 4.2,1.1 |
| Chin, P. T 2012 | 62 | 1.8,1.4* | 4.9,0.9 | 62 | 2.1,1.5 | 4.9,0.9 | 62 | 2.3,1.5 | 4.9,0.9 | 55 | 2.5,1.6 | 4.8,1.0 | 33 | 2.5,1.8 | 4.7,1.1 |
| Bozkurt, A. 2016 |  |  |  | 17 | 2.23,0.83 | 3.17,0.9 |  |  |  | 17 | 2.29,0.84 | 3.17,0.9 |  |  |  |

Supplement table 4 Qmax(ml/s)(mean,SD)

| Reference | 1m | | | 3m | | | 6m | | | 12m | | | 24m | | |
| --- | --- | --- | --- | --- | --- | --- | --- | --- | --- | --- | --- | --- | --- | --- | --- |
|  | N | Follow-up | Baseline | N | Follow-up | Baseline | N | Follow-up | Baseline | N | Follow-up | Baseline | N | Follow-up | Baseline |
| Sonksen, J 2015 |  |  |  | 33 | 13.6,5.3 | 9.4,3.5 | 33 | 13.5,5.5 | 9.6,3.4 | 32 | 13.6,5.5 | 9.6,3.5 |  |  |  |
| Sievert, K. D 2018 | 42 | 15.54,6.27 | 11.24,3.16 |  |  |  | 46 | 14.95,5.82 | 11.24,3.16 | 34 | 14.11,5.02 | 11.24,3.16 | 43 | 14.21,3.28 | 11.24,3.16 |
| Rukstalis, D 2016 |  |  |  | 42 | 11.95,5.79 | 7.95,2.45 |  |  |  | 43 | 12.07,5.28 | 8.09,2.50 | 36 | 12.18,5.76 | 8.00,2.55 |
| Rukstalis, D.2018 | 37 | 15,7.33 | 7.2,2.7 | 162 | 12.9,5.6 | 7.8,2.5 | 41 | 12.3,5.1 | 7.1,2.6 | 140 | 12.5,6.0 | 7.8,2.5 |  |  |  |
| Roehrborn,C.G2017 |  |  |  | 139 | 11.74,5.29 | 7.88,2.41 |  |  |  | 139 | 11.50,5.18 | 7.88,2.41 | 139 | `11.46,5.17 | 7.88,2.41 |
| Eure, G. R 2019 | 166 | 14.3,7.1 | 12.6,7.1 | 118 | 14.9,7.6 | 12.6,7.6 | 42 | 15.9,6.3 | 13.1,6.3 | 56 | 13.5,10.6 | 13.3,10.6 | 26 | 15.2,5.1 | 12.3,5.1 |
| Chin, P. T 2012 | 43 | 11.5,4.3* | 7.7,2.3 | 46 | 10.5,4.1 | 8.1,2.3 | 45 | 10.5,3.8 | 8.1,2.3 | 39 | 10.8,3.7 | 8.2,2.4 | 18 | 10.3,4.1 | 7.4,2.2 |
| Cantwell, A. L 2014 |  |  |  | 40 | 12.1,6.0 | 9.6,4.2 |  |  |  | 37 | 12.5,5.3 | 9.9,4.3 |  |  |  |
| Bozkurt, A. 2016 |  |  |  | 17 | 11.5,3.14 | 7.6,2.9 |  |  |  | 17 | 11.8,2.7 | 7.6,2.9 |  |  |  |

Supplement table 5 PVR(ml)(meanSD)

| Reference | 1m | | | 3m | | | 6m | | | 12m | | | 24m | | | |  |
| --- | --- | --- | --- | --- | --- | --- | --- | --- | --- | --- | --- | --- | --- | --- | --- | --- | --- |
|  | N | Follow-up | Baseline | N | Follow-up | Baseline | N | Follow-up | Baseline | N | Follow-up | Baseline | | N | Follow-up | Baseline | |
| Sonksen, J 2015 |  |  |  | 39 | 77.3,74.4 | 87.6,74. | 40 | 80.7,91.0 | 85.5,73.4 | 41 | 93.7,156.5 | 86.3,73.2 | |  |  |  | |
| Sievert, K. D 2018 | 41 | 91.05,235.06 | 149.5,251.4 |  |  |  | 41 | 50.8,61.1 | 149.5,251.4 | 33 | 63.0,44.2 | 149.5,251.4 | | 41 | 44.6,42.2 | 149.5,251.4 | |
| Rukstalis, D 2016 |  |  |  | 50 | 52.95,74.4 | 89.26,70.61 |  |  |  | 48 | 56.74,58.7 | 88.0,71.80 | | 42 | 79.23,69.11 | 86.55,72.71 | |
| Chin, P. T 2012 | 60 | 103,93 | 87,87 | 61 | 86,71 | 90,86 | 61 | 79,82 | 90,86 | 55 | 98,76 | 89,87 | | 31 | 89,104 | 54,68 | |
| Cantwell, A. L 2014 |  |  |  | 51 | 52.89,NA | 66.12,NA |  |  |  | 46 | 56.80,NA | 68.02,NA | |  |  |  | |
| Bozkurt, A. 2016 |  |  |  | 17 | 35.4,22.8 | 50.3,31.2 |  |  |  | 17 | 35.8,24.1 | 50.3,31.2 | |  |  |  | |

Supplement table 6 SHIM

| Reference | 1m | | | 3m | | | 6m | | | 12m | | | 24m | | |
| --- | --- | --- | --- | --- | --- | --- | --- | --- | --- | --- | --- | --- | --- | --- | --- |
|  | N | Follow-up | Baseline | N | Follow-up | Baseline | N | Follow-up | Baseline | N | Follow-up | Baseline | N | Follow-up | Baseline |
| Woo, H. H 2012 | 30 | 19.8,5.7 | 18.2,4.9 | 33 | 19.8,5.3 | 17.6,5.6 | 33 | 18.4,5.9 | 17.5,5.6 | 27 | 19.4,5.3 | 17.7,5.8 |  |  |  |
| Sonksen, J 2015 | 36 | 20.9,4.3 | 20.3，4.3 | 38 | 19.7,5.6 | 20.4,4.0 | 34 | 20.0,5.4 | 20.6,4.1 | 32 | 20.7,5.2 | 20.8,4.0 |  |  |  |
| Rukstalis, D 2016 | 34 | 16.15,8.08 | 15.65,7.85 | 37 | 16.51,8.17 | 16.46,7.26 | 37 | 16.51,8.17 | 16.46,7.26 | 38 | 16.55,7.68 | 16.05,7.56 | 34 | 17.79,6.68 | 17.12,6.79 |
| Rukstalis, D.2018 | 123 | 17.6,7.8 | 16.6,7.3 | 127 | 17.8,7.6 | 16.5,7.2 | 132 | 17.3,7.8 | 16.7,7.1 | 125 | 17.2,7.9 | 16.4,7.3 |  |  |  |
| McVary, K. T 2014 | 77 | 18.5,6.8 | 17.9,5.9 | 80 | 19.2,6.3 | 17.9,5.6 | 83 | 19.2,6.4 | 18.0,5.4 | 73 | 18.6,6.5 | 18.2,5.4 |  |  |  |
| Chin, P. T 2012 | 30 | 19.8,5.7* | 18.2,4.9 | 33 | 19.8,5.3 | 17.6,5.6 | 33 | 18.4,5.9 | 17.5,5.6 | 26 | 19.7,5.2 | 17.9,5.9 | 13 | 17.6,5.6 | 16.5,6.8 |
