# Supplementary material for: Urethral Lift as a Safe and Effective Procedure for Prostatic Hyplasia Population: A Systematic Review and Meta-Analysis
Source: Front Surg. 2020 Dec 8;7:598728. doi: 10.3389/fsurg.2020.598728 (PMC7793831; doi:10.3389/fsurg.2020.598728)
Supplement: Supplementary file 1 [file Data_Sheet_1.zip › Data Sheet 3.DOCX]

Supplement table 7 IPSS

| Item | 1m | 3m | 6m | 12m | 24m |
| --- | --- | --- | --- | --- | --- |
| Pool WMD | -11.287 | -11.605 | -11.014 | -10.143 | -9.396 |
| 95% Conf. Interval | -12.611;-10598 | -12.263;-9.830 | -11.937;-10.091 | -10.696;-9.590 | -10.267;-8.526 |
| Z&P value | 20.68(<0.001) | 22.60(<0.001) | 23.38(<0.001) | 35.93(<0.001) | 21.16(<0.001) |
| I^2^(%) | 78.0 | 72.1 | 56.3 | 0.0 | 0.0 |
| Research numbers | 10 | 10 | 10 | 11 | 5 |
| Patient numbers | 1443 | 1171 | 957 | 932 | 387 |

Supplement table 8 QoL

| Item | 1m | 3m | 6m | 12m | 24m |
| --- | --- | --- | --- | --- | --- |
| Pool WMD | -2.323 | -2.176 | -2.353 | -2.023 | -1.992 |
| 95% Conf. Interval | -2.630;-2.015 | -2.577;-1.775 | -2.633;-2.074 | -2.367;-1.678 | -2.205;-1.779 |
| Z&P value | 14.80(<0.001) | 1064(<0.001) | 14.49(<0.001) | 11.51(<0.001) | 18.36(<0.001) |
| I^2^ | 78.6 | 88.0 | 71.8 | 81.4 | 19.6 |
| Research numbers | 6 | 6 | 6 | 7 | 5 |
| Patient numbers | 1010 | 813 | 636 | 630 | 364 |

Supplement table 9 Qmax

| Item | 1m | 3m | 6m | 12m | 24m |
| --- | --- | --- | --- | --- | --- |
| Pool WMD | 4.420 | 3.653 | 3.545 | 3.582 | 3.391 |
| 95% Conf. Interval | 1.998;6.841 | 2.849;4.457 | 2.454;4.636 | 2.946;4.217 | 2.722;4.060 |
| Z&P value | 3.58(<0.001) | 8.90(<0.001) | 6.37(<0.001) | 11.04(<0.001) | 9.93(<0.001) |
| I^2^ | 85.6 | 53.5 | 40.4 | 26.3 | 0.0 |
| Research numbers | 4 | 8 | 4 | 9 | 5 |
| Patient numbers | 288 | 597 | 207 | 537 | 262 |

Supplement table 10 PVR

| Item | 1m | 3m | 6m | 12m | 24m |
| --- | --- | --- | --- | --- | --- |
| Pool WMD | -3.637 | -16.201 | -25.688 | -8.396 | -26.797 |
| 95% Conf. Interval | -67.939;60.665 | -28.702;-3.701 | -52.036;0.661 | -32.855;16.063 | -89.246;35.653 |
| Z&P value | 0.11(0.912) | 2.54(0.011) | 1.91(0.056) | 0.67(0.501) | 0.84(0.400) |
| I^2^ | 43.0 | 0.0 | 49.5 | 42.2 | 82.3 |
| Research numbers | 2 | 5 | 3 | 6 | 3 |
| Patient numbers | 101 | 218 | 142 | 240 | 114 |

Supplement table 11 SHIM

| Item | 1m | 3m | 6m | 12m | 24m |
| --- | --- | --- | --- | --- | --- |
| Pool WMD | 0.939 | 1.080 | 0.589 | 0.710 | 0.803 |
| 95% Conf. Interval | -0.003;1.880 | 0.157;2.003 | -0.344;1.522 | -0.266;1.686 | -1.859;3.464 |
| Z&P value | 1.95(0.051) | 2.29(0.022) | 1.24(0.216) | 1.43(0.154) | 0.59(0.554) |
| I^2^ | 0.0 | 0.0 | 0.0 | 0.0 | 0.0 |
| Research numbers | 6 | 6 | 6 | 6 | 2 |
| Patient numbers | 330 | 348 | 352 | 321 | 47 |
