# Supplementary material for: Urethral Lift as a Safe and Effective Procedure for Prostatic Hyplasia Population: A Systematic Review and Meta-Analysis
Source: Front Surg. 2020 Dec 8;7:598728. doi: 10.3389/fsurg.2020.598728 (PMC7793831; doi:10.3389/fsurg.2020.598728)
Supplement: Supplementary file 1 [file Data_Sheet_1.zip › Data Sheet 4.DOCX]

Supplement Table 11 IPSS

| Item | 1m | 3m | 6m | 12m | 24m |
| --- | --- | --- | --- | --- | --- |
| Fix mean | -10.707 | -11.048 | -10.936 | -10.1428 | -9.396 |
| Fix Conf. Interval | 11.001;10.412 | -11.376;-10.721 | -11.347;-10.1380 | -10.534;-9.752 | -10.012;-8.781 |
| Random mean | -11.319 | -11.629 | -11.054 | -10.201 | -9.396 |
| Random Conf. Interval | -12.363;-10.274 | -12.616;-10.643 | -11.970;-10.138 | -10.752;-9.651 | -10.012;-8.781 |
| test of Heterogeneity | 81.84(<0.001) | 64.55(<0.001) | 41.14(<0.001) | 17.16(0.071) | 2.81(0.591) |
| I^2^(%) | 81.9 | 86.1 | 78.1 | 41.7 | 0.0 |
| Research numbers | 10 | 10 | 10 | 11 | 5 |
| Patient numbers | 1443 | 1171 | 957 | 932 | 387 |

Supplement Table 12 QoL

| Item | 1m | 3m | 6m | 12m | 24m |
| --- | --- | --- | --- | --- | --- |
| Fix mean | -2.268 | -2.228 | -2.315 | -2.092 | -1.977 |
| Fix Conf. Interval | -2.3615;-2.174 | -2.351;-2.106 | -2.415;-2.215 | -2.191;-1.993 | -2.107;-1.847 |
| Random mean | -2.3264 | -2.160 | -2.360 | -2.009 | -2.019 |
| Random Conf. Interval | -2.630;-2.023 | -2.745;-1.574 | -2.637;-2.082 | -2.350;-1.668 | -2.239;-1.799 |
| test of Heterogeneity | 46.58(<0.001) | 100.31(<0.001) | 35.48(<0.001) | 64.54(<0.001) | 9.95(0.041) |
| I^2^(%) | 89.3 | 95.0 | 85.9 | 90.7 | 59.8 |
| Research numbers | 6 | 6 | 6 | 7 | 5 |
| Patient numbers | 1010 | 813 | 636 | 630 | 364 |

Supplement Table 13 Qmax

| Item | 1m | 3m | 6m | 12m | 24m |
| --- | --- | --- | --- | --- | --- |
| Fix mean | 3.764 | 3.870 | 3.443 | 3.655 | 3.391 |
| Fix Conf. Interval | 3.139;4.390 | 3.513;4.226 | 2.872;4.013 | 3.293;4.017 | 2.918;3.864 |
| Random mean | 4.495 | 3.588 | 3.575 | 3.468 | 3.391 |
| Random Conf. Interval | 2.081;6.909 | 2.791;4.385 | 2.478;4.672 | 2.817;4.120 | 2.918;3.864 |
| test of Heterogeneity | 41.76(<0.001) | 30.08(<0.001) | 13.42(0.009) | 21.69(<0.006) | 2.81(0.5894) |
| I^2^(%) | 92.8 | 76.7 | 70.2 | 63.1 | 0.0 |
| Research numbers | 4 | 8 | 4 | 9 | 5 |
| Patient numbers | 288 | 597 | 207 | 537 | 262 |

Supplement Table 14 PVR

| Item | 1m | 3m | 6m | 12m | 24m |
| --- | --- | --- | --- | --- | --- |
| Fix mean | 9.631 | -16.201 | -15.527 | -9.680 | -3.884 |
| Fix Conf. Interval | -12.158;31.420 | -25.040;-7.363 | -31.159;0.106 | -18.916;-0.444 | -20.675;12.906 |
| Random mean | -12.431 | -16.383 | -28.755 | -9.827 | -20.675 |
| Random Conf. Interval | -83.325;58.464 | -29.036;-3.731 | -66.951;9.441 | -27.035;7.381 | -79.715;38.364 |
| test of Heterogeneity | 3.51(0.061) | 5.59(0.133) | 9.33(0.009) | 10.19(0.037) | 19.05(<0.001) |
| I^2^(%) | 71.5 | 46.3 | 78.6 | 60.8 | 89.5 |
| Research numbers | 2 | 5 | 3 | 6 | 3 |
| Patient numbers | 101 | 218 | 142 | 240 | 114 |

Supplement Table 15 SHIM

| Item | 1m | 3m | 6m | 12m | 24m |
| --- | --- | --- | --- | --- | --- |
| Fix mean | 0.938 | 1.081 | 0.589 | 0.710 | 0.794 |
| Fix Conf. Interval | 0.272;1.605 | 0.428;1.733 | -0.071;1.248 | 0.020;1.401 | -2.895;4.484 |
| Random mean | 0.938 | 1.080 | 0.589 | 0.710 | 0.794 |
| Random Conf. Interval | 0.272;1.605 | 0.190;1.970 | -0.071;1.248 | 0.020;1.401 | -2.895;4.484 |
| test of Heterogeneity | 1.48(0.916) | 8.74(0.120) | 3.35(0.646) | 3.07(0.689) | 0.01(0.918) |
| I^2^(%) | 0.0 | 42.8 | 0.0 | 0.0PVR | 0.0 |
| Research numbers | 6 | 6 | 6 | 6 | 2 |
| Patient numbers | 330 | 348 | 352 | 321 | 47 |
