# Supplementary material for: Urethral Lift as a Safe and Effective Procedure for Prostatic Hyplasia Population: A Systematic Review and Meta-Analysis
Source: Front Surg. 2020 Dec 8;7:598728. doi: 10.3389/fsurg.2020.598728 (PMC7793831; doi:10.3389/fsurg.2020.598728)
Supplement: Supplementary file 1 [file Data_Sheet_1.zip › Data Sheet 5.DOCX]

IPSS

| Item | 1m | 3m | 6m | 12m | 24m |
| --- | --- | --- | --- | --- | --- |
| Fix mean | 11.096 | 11.040 | 10.9413 | 11.954 | 12.087 |
| Fix Conf. Interval | 10.794；11.398 | 10.704;11.376 | 10.492;11.390 | 11.548;12.359 | 11.473;12.701 |
| Random mean | 11.145 | 11.042 | 11.074 | 12.117 | 12.991 |
| Random Conf. Interval | 10.253；12.037 | 10.236;11.849 | 10.441;11.708 | 11.392;12.842 | 10.991;13.998 |
| test of Heterogeneity | 52.71(<0.001) | 38.84(<0.001) | 16.16(0.064) | 27.31(0.002) | 20.73(<0.001) |
| I^2^(%) | 82.9 | 76.8 | 44.3 | 63.4 | 80.7 |
| n | 1443 | 1171 | 957 | 932 | 387 |

Qol

| Item | 1m | 3m | 6m | 12m | 24m |
| --- | --- | --- | --- | --- | --- |
| Fix mean | 2.227 | 2.160 | 2.208 | 2.350 | 2.382 |
| Fix Conf. Interval | 2.134;2.330 | 2.067;2.254 | 2.098;2.318 | 2.241;2.458 | 2.246;2.517 |
| Random mean | 2.252 | 2.160 | 2.208 | 2.350 | 2.410 |
| Random Conf. Interval | 2.032;2.472 | 2.067;2.254 | 2.098;2.318 | 2.241;2.458 | 2.146;2.673 |
| test of Heterogeneity | 18.53(0.002) | 4.44(0.489) | 2.90(0.716) | 3.47(0.748) | 12.69(<0.013) |
| I^2^(%) | 73.0 | 0.0 | 0.0 | 0.0 | 68.5 |
| n | 1010 | 813 | 636 | 630 | 364 |

Qmax

| Item | 1m | 3m | 6m | 12m | 24m |
| --- | --- | --- | --- | --- | --- |
| Fix mean | 13.728 | 12.310 | 12.659 | 12.076 | 12.615 |
| Fix Conf. Interval | 13.006;14.450 | 11.873;12.746 | 11.975;13.344 | 11.638;12.514 | 12.058;13.173 |
| Random mean | 14.132 | 12.373 | 13.331 | 12.255 | 12.670 |
| Random Conf. Interval | 12.191;16.073 | 11.431;13.316 | 11.284;15.378 | 11.584;12.926 | 11.004;14.336 |
| test of Heterogeneity | 18.81(<0.001) | 29.38(<0.001) | 33.49(<0.001) | 16.52(0.036) | 29.73(<0.001) |
| I^2^(%) | 84.0 | 76.2 | 88.1 | 51.6 | 86.5AS |
| n | 288 | 597 | 207 | 537 | 262 |

SHIM

| Item | 1m | 3m | 6m | 12m | 24m |
| --- | --- | --- | --- | --- | --- |
| Fix mean | 19.004 | 18.893 | 18.407 | 18.625 | 17.723 |
| Fix Conf. Interval | 18.311;19.697 | 18.214;19.573 | 17.706;19.108 | 17.909;19.342 | 15.916;19.530 |
| Random mean | 18.906 | 18.910 | 18.401 | 18.707 | 17.723 |
| Random Conf. Interval | 17.589;20.224 | 17.989;19.830 | 17.433;19.369 | 17.494;19.921 | 15.916;19.530 |
| test of Heterogeneity | 16.82(0.005) | 8.69(0.122) | 8.89(0.114) | 13.63(0.018) | 0.01(0.921) |
| I^2^(%) | 70.3 | 42.4 | 43.7 | 63.3 | 0.0 |
| N | 330 | 348 | 352 | 321 | 47 |

PVR

| Item | 1m | 3m | 6m | 12m | 24m |
| --- | --- | --- | --- | --- | --- |
| Fix mean | 101.845 | 52.910 | 66.884 | 59.913 | 57.050 |
| Fix Conf. Interval | 79.479;124.211 | 44.967;60.853 | 54.460;79.309 | 52.572;67.254 | 46.525;67.574 |
| Random mean | 10.845 | 62.183 | 68.814 | 70.848 | 68.083 |
| Random Conf. Interval | 79.479;124.211 | 35.880;88.488 | 48.415;89.213 | 46.461;95.235 | 38.858;97.309 |
| test of Heterogeneity | 0.10(0.757) | 27.47(<0.001) | 5.09(0.078) | 36.85(<0.001) | 10.82(0.005) |
| I^2^(%) | 0.0 | 89.1 | 60.7 | 89.1 | 51.5 |
| n | 101 | 218 | 142 | 240 | 114 |

IPSS-baseline

| Item | 1m | 3m | 6m | 12m | 24m |
| --- | --- | --- | --- | --- | --- |
| Fix mean | 22.005 | 22.124 | 22.115 | 22.226 | 22.051 |
| Fix Conf. Interval | 21.724;22.285 | 21.819;22.428 | 21.756;22.474 | 21.873;22.579 | 21.488;22.614 |
| Random mean | 22.201 | 22.666 | 22.295 | 22.428 | 22.159 |
| Random Conf. Interval | 21.801;23.202 | 21.926;23.406 | 21.300;23.290 | 21.741;23.116 | 21.002;23.317 |
| test of Heterogeneity | 41.19(<0.001) | 42.63(<0.001) | 64.15(<0.001) | 32.94(<0.001) | 14.22(0.007) |
| I^2^(%) | 78.1 | 78.9 | 86.0 | 69.6 | 71.9 |
| n | 1443 | 1171 | 957 | 932 | 387 |

QoL-baseline

| Item | 1m | 3m | 6m | 12m | 24m |
| --- | --- | --- | --- | --- | --- |
| Fix mean | 4.585 | 4.415 | 4.587 | 4.513 | 4.491 |
| Fix Conf. Interval | 4.506;4.664 | 4.341;4.489 | 4.505;4.669 | 4.430;4.597 | 4.378;4.603 |
| Random mean | 4.584 | 4.386 | 4.568 | 4.381 | 4.487 |
| Random Conf. Interval | 4.327;4.841 | 3.999;4.772 | 4.342;4.794 | 4.083;4.679 | 4.237;4.737 |
| test of Heterogeneity | 48.42(<0.001) | 119.81(<0.001) | 34.23(<0.001) | 67.85(<0.001) | 17.25(0.002) |
| I^2^(%) | 89.7 | 95.8 | 85.4 | 91.2 | 76.893. |
| n | 1010 | 813 | 636 | 630 | 364 |

Qmax-baseline

| Item | 1m | 3m | 6m | 12m | 24m |
| --- | --- | --- | --- | --- | --- |
| Fix mean | 9.076 | 8.113 | 8.878 | 8.219 | 8.342 |
| Fix Conf. Interval | 8.645;9.507 | 7.886;8.340 | 8.471;9.286 | 7.986;8.453 | 8.026;8.657 |
| Random mean | 9.664 | 8.709 | 9.712 | 8.935 | 9.213 |
| Random Conf. Interval | 7.202;12.127 | 7.983;9.435 | 7.909;11.516 | 8.182;9.688 | 7.720;10.706 |
| test of Heterogeneity | 93.84(<0.001) | 55.16(<0.001) | 70.37(<0.001) | 62.15(<0.001) | 60.88(<0.001) |
| I^2^(%) | 96.8 | 87.3 | 94.3 | 78.1 | 93.4 |
| n | 288 | 597 | 207 | 537 | 262 |

SHIM-baseline

| Item | 1m | 3m | 6m | 12m | 24m |
| --- | --- | --- | --- | --- | --- |
| Fix mean | 18.024 | 17.985 | 17.973 | 18.081 | 16.949 |
| Fix Conf. Interval | 17.389;18.659 | 17.371;18.599 | 17.362;18.584 | 17.430;18.732 | 15.007;18.891 |
| Random mean | 17.930 | 17.822 | 17.871 | 17.919 | 16.949 |
| Random Conf. Interval | 16.686;19.175 | 16.507;19.137 | 16.588;19.155 | 16.412;19.427 | 15.007;18.891 |
| test of Heterogeneity | 17.99(0.003) | 21.22(<0.001) | 20.28(0.001) | 24.33(<0.001) | 0.08(0.7797) |
| I^2^(%) | 72.2 | 76.4 | 75.3 | 79.5 | 0.0 |
| n | 330 | 348 | 352 | 321 | 47 |

PVR-baseline

| Item | 1m | 3m | 6m | 12m | 24m |
| --- | --- | --- | --- | --- | --- |
| Fix mean | 91.728 | 73.253 | 90.318 | 73.029 | 79.954 |
| Fix Conf. Interval | 70.563；112.893 | 63.786;82.720 | 74.976;105.660 | 63.489;82.568 | 59.107;90.801 |
| Random mean | 106.927 | 78.363 | 91.109 | 81.430 | 82.816 |
| Random Conf. Interval | 49.840；146.014 | 56.179;100.548 | 73.086;109.133 | 59.138;103.722 | 46.311;119.320 |
| test of Heterogeneity | 2.34(0.126) | 15.55(0.001) | 2.45(0.294) | 17.36(0.002) | 7.62(0.022) |
| I^2^(%) | 57.3 | 80.7 | 18.2 | 77.0 | 73.7 |
| n | 101 | 218 | 142 | 240 | 114 |
